# CS-SELEX Generates High-Affinity ssDNA Aptamers as Molecular Probes for Hepatitis C Virus Envelope Glycoprotein E2

**DOI:** 10.1371/journal.pone.0008142

**Published:** 2009-12-03

**Authors:** Fang Chen, Yilan Hu, Dongqing Li, Haidan Chen, Xiao-Lian Zhang

**Affiliations:** State Key Laboratory of Virology, Department of Immunology, Hubei Province Key Laboratory of Allergy and Immunology, Wuhan University School of Medicine, Wuhan, People's Republic of China; Charité-Universitätsmedizin Berlin, Germany

## Abstract

Currently, the development of effective diagnostic reagents as well as treatments against Hepatitis C virus (HCV) remains a high priority. In this study, we have described the development of an alive cell surface -Systematic Evolution of Ligands by Exponential Enrichment (CS-SELEX) technique and screened the functional ssDNA aptamers that specifically bound to HCV envelope surface glycoprotein E2. Through 13 rounds of selection, the CS-SELEX generated high-affinity ssDNA aptamers, and the selected ssDNA aptamer ZE2 demonstrated the highest specificity and affinity to E2-positive cells. HCV particles could be specifically captured and diagnosed using the aptamer ZE2. A good correlation was observed in HCV patients between HCV E2 antigen-aptamer assay and assays for HCV RNA quantities or HCV antibody detection. Moreover, the selected aptamers, especially ZE2, could competitively inhibit E2 protein binding to CD81, an important HCV receptor, and significantly block HCV cell culture (HCVcc) infection of human hepatocytes (Huh7.5.1) in vitro. Our data demonstrate that the newly selected ssDNA aptamers, especially aptamer ZE2, hold great promise for developing new molecular probes, as an early diagnostic reagent for HCV surface antigen, or a therapeutic drug specifically for HCV.

## Introduction

Hepatitis C virus (HCV) is estimated to have infected 3% of the world's population (approximately 170 million people). Approximately 80% of the infected patients develop liver cirrhosis and, in some cases, hepatocarcinoma [1], [2]. Recent reports have shown more HCV recurrence and decreased survival for HCV following orthotopic liver transplantation or insulin resistance in patients with chronic hepatitis C [3], [4]. In addition, there is a high prevalence of HCV, HIV, and hepatitis B virus (HBV) coinfection, which requires that the access to diagnosis and therapies for these infections is improved. Presently, the only available therapy is alpha interferon (IFN-α) alone or in combination with ribavirin [5]–[7]. Such treatments are expensive, show low response rates, and carry the risk of significant side effects [6]–[8]. Effective therapies to counteract this important public health problem are still lacking.

Early diagnosis of HCV infection in HCV- or HIV-infected patients has significant implications for patient management. However, the currently recommended serological screening strategy for identifying anti-HCV antibodies is unable to detect plasma donations that are anti-HCV-negative and HCV RNA-positive during the pre-seroconversion window period (PWP). Another method for detecting HCV RNA is reverse transcript polymerase chain reaction (RT-PCR), which is not feasible due to its high cost; it is not used routinely in the diagnostic laboratories, especially in developing countries [9], [10]. The latest breakthrough in diagnosing early HCV infection is by detecting the HCV core antigen that is present during the early stage or before seroconversion [11]. However, relying solely on a single HCV core antigen assay may not be useful for a definite diagnosis of early HCV infection. None of the current methods are available to measure the HCV surface antigens in serum. More sensitive and less expensive assays for the early diagnosis of HCV are needed.

HCV is a member of the Flaviviridae family and is an enveloped virus with a single-stranded RNA genome approximately 9.5 kb in length that contains a single open reading frame [12]–[14]. The open reading frame encodes a polyprotein of approximately 3,010 amino acids that is processed into at least 10 mature proteins (C, E1, E2, p7, NS2, NS3, NS4A, NS4B, NS5A, and NS5B) by both host signal peptidases and viral proteases. HCV contains two heavily glycosylated envelope glycoproteins, E1 and E2. E2 is thought to initiate viral attachment [15], whereas E1 may be involved in virus-cell membrane fusion. The envelope E2 glycoprotein plays a critical role in the initiating infection through recognizing and binding to human cellular receptors [16]–[20]. It has been proposed that the E2 glycoprotein binds to CD81, a tetraspanin molecule that is expressed on hepatocytes and B lymphocytes. HCV infection is dependent on at least three co-receptors: CD81, scavenger receptor BI (SR-BI), and claudin-1 [16]–[20]. Among them, CD81 has been identified as a critical co-receptor for HCV particle entry [16]–[20].

High affinity aptamers for specific target molecules can be isolated from a library of randomized sequences *in vitro* using the SELEX (systematic evolution of ligands by exponential enrichment) process [21]. SELEX involves sequential rounds of selection and amplification from a vast pool of nucleic acids (10^14^ molecules) for ligands (aptamers) that bind with high affinity to target molecules. Aptamers are short, single-stranded oligonucleotides that can fold into specific three-dimensional structures in order to recognize target molecules, such as small chemicals, proteins, or even cells; as such, they have several potential advantages over antibodies and antibiotics. Being smaller than antibodies, aptamers are better candidates for cell penetration and blood clearance. Aptamers have been used for pure recognition, inhibition, diagnostic, and therapeutic applications [21].

Small aptamers that inhibit the virus at the stage of viral entry (e.g., by blocking the interactions between the viral envelope glycoprotein and the cellular receptor or co-receptor) have not been described so far. Several aptamers have been selected against HCV [22]–[24]. However, none of these have been selected against surface envelope glycoproteins. Currently, few SELEX strategies exist to generate inhibitors against glycosylated viral envelope proteins.

In the present study, we developed a new SELEX procedure termed alive cell surface-SELEX (CS-SELEX), which targets the E2 envelope glycoprotein expressed on the mammalian cell line CT26. In this model system, we carried out CS-SELEX by establishing a stable cell line that ectopically expressed the HCV E2 glycoprotein on the cell surface and examined the binding affinities and functions of selected ssDNA aptamers.

## Results

### High-affinity Aptamers for HCV-E2 Glycoprotein Were Isolated by CS-SELEX

**Figure 1:**
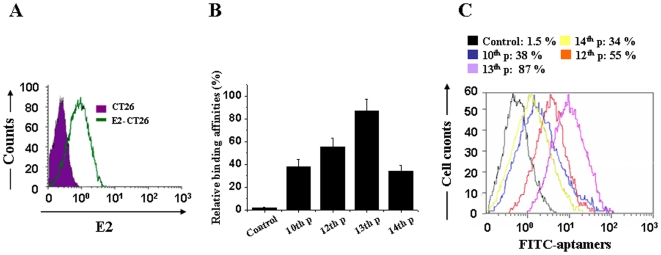
High-affinity aptamers for HCV-E2 glycoprotein were isolated by CS-SELEX. (A) The E2 stably expressing cell line E2-CT26 was established, and E2-CT26 E2 cell surface expression was identified by a PE-conjugated anti-E2 antibody using flow cytometry. (B) Comparison of binding percentages of different pools of FITC-labeled aptamers with E2-CT26 cells using flow cytometry. Data shown were calculated as mean±SEM, and data are from three independent experiments. (C) Representative results from different pools of FITC-labeled aptamers binding with E2-CT26 cells by flow cytometry.

To isolate aptamers that specifically bind to the HCV E2 envelope glycoprotein, we utilized E2-expressing CT26 cells as a selection target; CT26 cells were used for counter selection. Our approach relies on CS-SELEX to evolve aptamers for whole live cells that express a variety of surface markers representing molecular differences among E2-CT26 cells and CT26 cells. The random ssDNA library (ca.10^14^ molecules) was used to screen ligands that bind to E2-CT26 cells (Fig. 1A). Aptamers that bind to E2-CT26 cells with high affinity and specificity were successfully obtained.

After 14 rounds of selection, different pools of aptamers were obtained using CS-SELEX, and the binding affinities between aptamers and E2-CT26 cells were measured by flow cytometry as described in the Methods. The sequence of binding affinities between FITC-labeled aptamers was as follows: 13^th^ Pool>12^th^ Pool>10^th^ Pool>14^th^ Pool>1^st^ Pool, as shown in Figs. 1B and 1C.

The 13^th^ Pool clones were cloned into pUC19, and individual clones of single aptamers were selected and sequenced. The consensus regions within the individual aptamers are shown in Table 1. The single aptamer ZE2 has the highest binding affinity among all aptamers with a Kd of 1.05±1 nM. The sequence of binding affinities was as follows: ZE2 (Kd 1.05±1 nM)>13^th^ P (Kd 1.67±0.9 nM)>ZE3 (Kd 3.22±0.7 nM)>12^th^ P (Kd 6.34±0.9 nM)>ZE5 (Kd 4.29±0.6 nM)>ZE4 (Kd 8.85±1 nM)>10^th^ P (Kd 16.6±6 nM) (Table 2).

**Table 1:** Consensus regions within the representive individual aptamers are underlined or shaded.

Aptamer	Frequency	ssDNA aptamer sequences (N_30_)
ZE1	1/8	ATAGGGCACTTGTCTCACACCACGGTGTGA
ZE2	2/8	GAATGAGGAATAATCTAGCTCCTTCGCTGA
ZE3	2/8	CGCCGTATTAAGATTGGGAGACCTGGTAGA
ZE4	1/8	GGGCCTCGATTTAGTTCGCGGCCATAGGGC
ZE5	1/8	TCCATTCATGTAACGAACATAGTTTTGGCA
ZE6	1/8	AGTTCGACTCCGTTAGGTGTCGCTGTAGGT
Consensus		-A-T----T-

**Table 2:** Binding affinities or Kd values of different aptamers.

Aptamers	4^th^ P	10^th^ P	12^th^ P	13^th^ P	14^th^ P	ZE2	ZE3	ZE5
Kd (nM)	51.4±7	16.6±6	6.34±5.5	1.67±0.9	50.3±13	1.05±0.4	3.22±0.7	4.29±0.6

“P” represents pool; ZE2, ZE3, and ZE5 are different single aptamers from the 13th pool.

### The SsDNA Aptamer ZE2 with the Highest Binding Affinity Specifically Targets HCV E2

**Figure 2:**
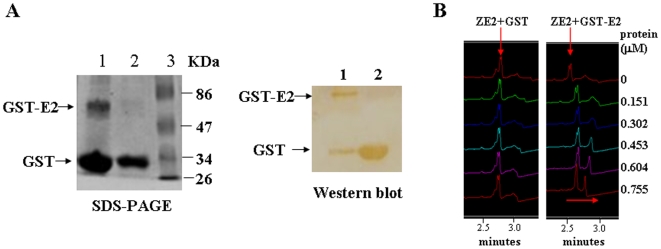
Selected aptamers specifically bind to HCV E2. (A) SDS-PAGE (Left) and western blot analysis (Right) of the purified GST-E2 and GST recombinant proteins. (B) Capillary electrophoresis analysis of single aptamer ZE2 binding to different doses of GST-E2 protein. Representative results of CEMSA showed that the binding peak of ZE2 with GST-E2 migrated from left to right, but the binding peak of ZE2 with GST had no change.

The single aptamer ZE2 was chosen for further characterization, as it had the highest E2 binding affinity. Firstly, the interaction between the ssDNA aptamer ZE2 and the E2 protein was demonstrated by capillary zone electrophoresis. The GST-E2 and GST recombinant proteins were purified and confirmed using SDS-PAGE and western blot analysis (Fig. 2A). The capillary electrophoresis method is based on simple UV detection at 260 nm with a linear polymer buffer and a coated capillary; it requires no labeling or derivatization of the DNA. We observed that the DNA aptamer ZE2 was facilely adsorbed to the GST-E2 protein but not to the GST protein (Fig. 2C), and the binding was completed within 10 min. A specific E2 protein-DNA aptamer ZE2 complex was observable as a retarded peak, which increased with increasing protein concentration with a corresponding reduction in the free DNA peak (Fig. 2B).

### Fluorescence Microscope Imaging of E2-expressing Cells with FITC-ZE2 Aptamer

**Figure 3:**
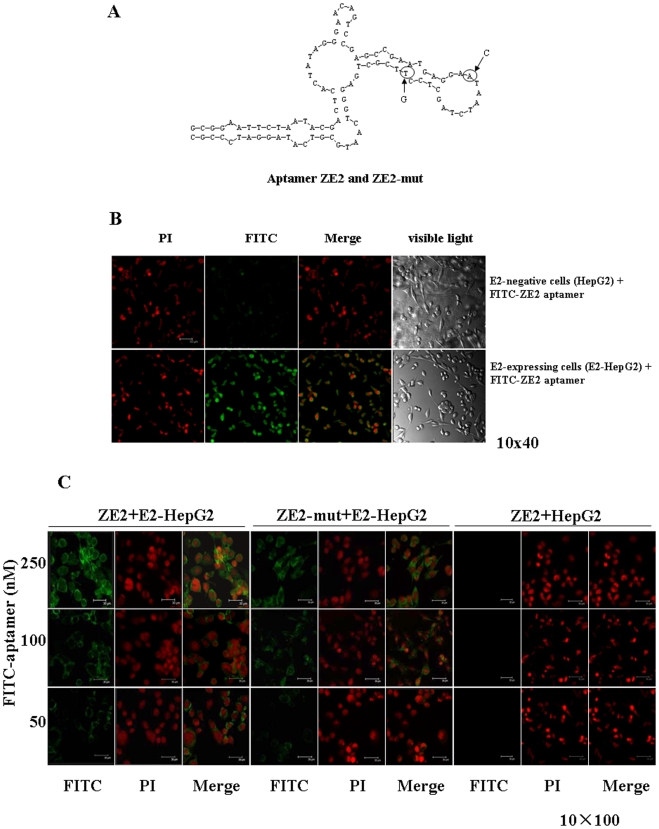
The characterization of aptamer ZE2 specificity for E2-expressing cells. (A) Prediction of ZE2 secondary structure. Arrows represent exchanged bases in ZE2-mut compared to that of the ZE2 aptamer. (B) Fluorescence microscope imaging of E2-expressing cells with FITC-ZE2. FITC-ZE2 bound to E2-HepG2 cells but not to HepG2 cells by confocal immunofluorescence microscopy. (C) Comparison of different doses of FITC-ZE2 or FITC-ZE2-mut binding to the E2-HepG2 or HepG2 cells by confocal immunofluorescence microscopy.

The secondary structure of the ssDNA aptamer ZE2 was predicted with the DNAMAN software (Lynnon Biosoft), and it showed that the terminal loop of a stem-loop structure might be the site of binding to the target E2 protein (Fig. 3A). To test this possibility, a ssDNA aptamer ZE2-mut was synthesized, in which two bases in the terminal loop of a stem-loop structure of ZE2 were mutated (Fig. 3A). We further observed that E2-expressing cells, E2-HepG2, specifically bound FITC-ZE2 aptamers under the fluorescence microscope (Figs. 3B and 3C). The fluorescence intensities of the binding affinities between FITC-ZE2 and E2-expressing cells were aptamer dose-dependent (Fig. 3C), and a much smaller fluorescence intensity was observed when using the FITC-ZE2-mut (Fig. 3C). Similar results were observed for fluorescence microscope imaging of E2-CT26 cells bound with the FITC-ZE2 aptamer (data not shown). No binding between FITC-ZE2 and HepG2 or CT26 cells was detected (Figs. 3B and 3C).

The above data suggest that the ssDNA aptamer ZE2 specifically targets HCV envelope glycoprotein E2-expressing cells, and FITC-ZE2 can be used as a probe to detect E2-expressing cells using a fluorescence microscope.

### The ssDNA Aptamer ZE2 Specifically Targets HCV Particles

**Figure 4:**
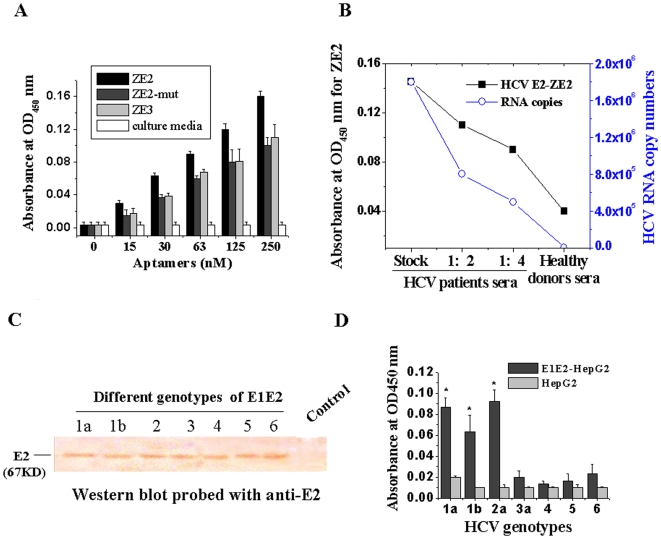
The ssDNA aptamer ZE2 specifically targets HCV particles. (A) HCVcc could be captured by the aptamer ZE2 in an aptamer dose-dependent manner by sandwich ELISA. Plates were coated with anti-E2 polyclonal antibody and blocked with 1% BSA. After extensive washing, the bound viral particles were added and then revealed using biotin-labeled aptamer ZE2, ZE3 or ZE2-mut as described in Materials and Methods. Data shown were calculated as mean±SEM, and data are from three independent experiments. (B) Comparison of HCV detection methods: HCV E2 antigen-aptamer method and HCV RNA quantification by real time fluorescence quantitative RT-PCR. The aptamer method was performed as above (A), except HCV patients and healthy donor serum samples were added into each 96-well ELISA plate instead of HCVcc. (C) Determination of E2 protein expressions of different genotypes of E1E2 gene stable-expressing HepG2 cells with anti-E2 antibody by western blot analysis. HepG2 was uesd as a control. (D) Different genotypes of E2 detected by aptamer ZE2. Data shown were calculated as mean±SEM and data are from three independent experiments.

To determine whether this selected aptamer ZE2 could bind to HCV particles, virus capture assays were performed by sandwich ELISA. Virus samples from HCVcc were incubated in the anti-E2 polyclonal antibody-precoated ELISA plates. Biotin-aptamers were then added and incubated, followed by extensive washing to remove unbound aptamers. The bound viral particles were revealed by the biotin-labeled aptamers as described in the Materials and Methods. We found that ZE2 aptamer binding to the HCV particles was aptamer dose-dependent, but the ZE3 aptamer or the ZE2-mut with two mutated bases had a much lower binding affinity (Fig. 4A). These results are consistent with their binding affinities with E2 glycoprotein. No binding was observed between the ZE2 aptamer and control culture media (Fig. 4A).

In addition, sera samples from 154 Chinese HCV patients (both HCV antibody- and HCV RNA-positive) and 155 healthy donors (both HCV antibody- and HCV RNA- negative) were collected and measured using the biotin-ZE2 probe with sandwich ELISA. As shown in Table 3 and Fig. 4B, a good correlation was observed between HCV E2 antigen-aptamer assay and assays for HCV RNA quantities or HCV antibody detection. Biotin-labeled ZE2 could bind to HCV particles in HCV patient samples with much higher affinities than to healthy donor samples (* *p*<0.05, Table 3, Fig. 4B), and the absorbance values of the HCV serum samples were not only proportional to serial dilutions of sera, but also to HCV RNA copies measured by RT-PCR (Fig. 4B).

**Table 3:** Comparison of HCV E2 antigen-aptamer ZE2 assay and assays for HCV RNA quantities or HCV antibody detection.

Characteristic	Healthy individuals (N = 155)	HCV patients (N = 154)	*p*-Value
Age-yr	41.1±11.2	45.5±13.4	0.85
Male sex-no. (%)	75(80)	76(78)	0.83
Race or ethnic group-no. (%) Asian	155 (100)	154 (100)	1.00
Region-no. (%) Hubei province, China	155 (100)	154 (100)	1.00
Anti-HCV-positive-no. (S/CO)(%)	<1 (0)	>1 (100)	<0.001
HCV RNA –IU/ml (%)	<50 (0)	>500 (100)	<0.001
OD450 (ZE2 binding for HCV)	0.04±0.005	0.13±0.06	<0.05

Cutoff value (CO.) = 0.1x mean OD_450_ of positive samples + mean OD_450_ of negative controls.

Anti-HCV-positive: OD_450_ of sample >CO., or S (Sample)/CO >1, anti-HCV-negative: OD_450_ of sample <CO., or S/CO<1.

These data suggest that the ssDNA aptamer ZE2 specifically targets HCV particles and could be used to diagnose early HCV infection by detecting HCV surface antigen in sera that is present during the early stage or before seroconversion.

### Genotypes 1a, 1b, and 2a of E2 Can be Significantly Captured by Aptamer ZE2

HCV has six major genotypes and numerous subtypes based on its positive-strand RNA genome, and a seventh major genotype was recently discovered [25]. E1E2 gene (from genotypes 1 to 6) stable-expressing HepG2 cells were established by G418 selection, and E2 expressions from these cells were determined by western blot analysis (Fig. 4C). Different genotypes of E1E2 gene stable-expressing HepG2 cells were used to coat ELISA plates. We utilized ELISA to determine which genotypes of E1E2 could be bound by ZE2. The results showed that aptamer ZE2 was specifically targeted to the E2 proteins of genotypes 1a, 1b, and 2a, and the binding affinities of aptamer ZE2 with genotypes 1a, 1b, and 2a of E2 were three to five-fold higher than those of genotypes 3, 4, 5, and 6 (Fig. 4D).

### Selected Aptamers Block E2 Protein Binding to Target Huh7.5.1 Cells

**Figure 5:**
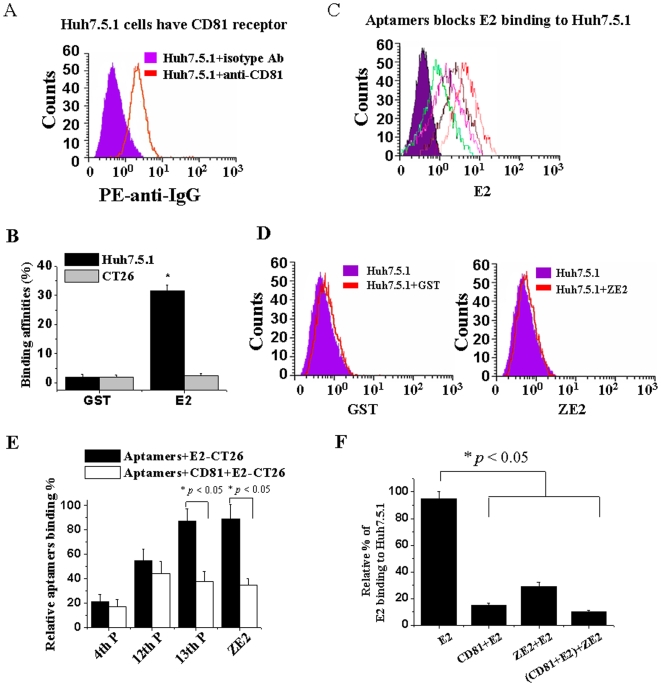
DNA aptamer ZE2 and viral receptor CD81 share similar binding sites on HCV E2. (A) CD81 molecules are expressed on the cellular surface of hepatocytes Huh7.5.1; flow cytometric analysis with PE-labeled anti-CD81 antibody. PE-conjugated rat IgG1 was used as an isotype-matched control antibody (Ab). (B) HCV E2 had a much higher binding affinity for Huh7.5.1 than CT26 cells by flow cytometric analysis. E2-GST or GST proteins were preincubated with 1×10^6^ Huh7.5.1 or CT26 cells, respectively. Anti-GST antibody and FITC-anti-IgG were added and analyzed by flow cytometric analysis. (C) Different pools of aptamers or single DNA aptamer ZE2 can block HCV E2 protein binding to Huh7.5.1 cells. (D) GST and ZE2 do not bind to huh7.5.1 cells by flow cytometric analysis. (E) CD81 competitively blocked FITC-aptamer binding to E2-CT26 cells by flow cytometric analysis. (F) Both ZE2 and CD81 competitively blocked E2 binding to Huh7.5.1 cells, as determined by flow cytometry with FITC-conjugated anti-E2 antibody. All data are mean±SEM from six separate experiments.

As the aptamer ZE2 specifically binds to HCV E2, we next further determined whether aptamer ZE2 also has the ability to block HCV E2 binding to its target cells. Human hepatocyte Huh7.5.1 cells are supposed to express the HCV E2-binding receptor CD81, and human CD81 has been described as a main receptor for HCV (16–20). We used a PE-labeled anti-CD81 antibody to detect whether Huh7.5.1 cells expressed CD81. A flow cytometric analysis showed that PE-labeled anti-CD81 antibody bound to Huh7.5.1 cells with a much higher binding affinity than that of the PE-labeled control antibody (Ab) (Fig. 5A). When the E2 or GST proteins were added, Huh7.5.1 cells significantly bound to the E2 protein (Fig. 5B), and their binding affinities were much higher than they were for the control GST protein (Fig. 5B, * *p*<0.05). The E2 protein specifically bound to Huh7.5.1 cells but not to CT26 cells (Fig. 5B). All of the above data suggest that Huh7.5.1 cellular surfaces have HCV E2-binding CD81 receptor molecules.

The flow cytometric experiments revealed that the selected aptamers significantly blocked HCV E2 protein binding to Huh7.5.1 cells (Fig. 5C). The sequence of inhibition effects was as follows: ZE2>13^th^ P>6^Th^ P (Fig. 5B). The inhibitory effects of the aptamers were consistent with their binding affinity results shown in Table 1. However, GST and ZE2 had no significant binding affinities for Huh7.5.1 cells (Fig. 5D). The recombinant CD81 protein could competitively inhibit the aptamers from binding to the E2-expressing cells (E2-CT26), especially ZE2 and the 13^th^ pool aptamers (Fig. 5E), while no or weak inhibitory effects were observed for the 4^th^ and 12^th^ pool aptamers (Fig. 5E), which due to their less binding affinities to E2 comparing the 13^th^ pool aptamers and ZE2 aptamer (Table 2). Moreover, ZE2 or CD81 blocked approximately 80% of HCV E2 protein binding to CD81-expressing Huh7.5.1 cells, respectively (Fig. 5F). The addition of both ZE2 and CD81 blocked approximately 90% of HCV E2 protein binding to Huh7.5.1 cells (Fig. 5F). All of these data indicate that ZE2 and CD81 may share similar binding sites on HCV E2 and that aptamer ZE2 can block HCV E2 binding to target Huh7.5.1 cells.

### Aptamer ZE2 Dramatically Inhibits HCV Infection of Huh7.5.1 Cells

**Figure 6:**
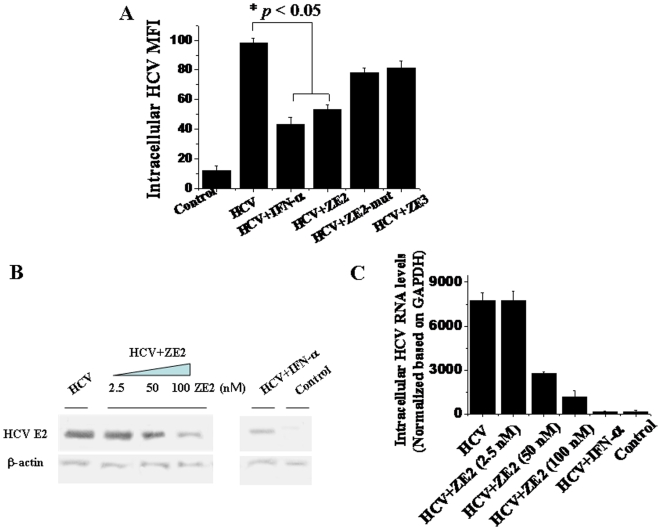
Aptamer ZE2 dramatically blocks HCVcc infection of hepatocytes. (A) Immunofluorescent detection of intracellular HCV mean fluorescence density (MFI) of HCVcc infected Huh-7.5.1 cells with anti-E2 antibody. The data shown were calculated as mean±SEM and data are from three independent experiments. (B) Western blot analysis of intracellular E2 protein expressions of HCVcc infected Huh7.5.1 in the presence of ZE2 or IFN-α with specific anti-E2 antibody; β-actin, a housekeeping gene with constant expression, was used as internal control. β-actin protein was detected with specific anti- β-actin antibody. (C) Quantitation of intracellular HCV RNA was detected using real-time quantitative RT-PCR and normalized to the levels of GAPDH mRNA. GAPDH, a housekeeping gene with constant mRNA expression, was used as internal control. The data shown were calculated as mean±SEM and data are from three independent experiments.

Finally, we examined the inhibitory effects of aptamer ZE2 on HCV infection of human hepatocyte Huh7.5.1 cells. Immunofluorescence microscopy demonstrated that the mean fluorescence intensities (MFIs) of intracellular HCV were significantly decreased upon the addition of the ZE2 aptamer or IFN-α (Fig. 6A). While the ZE2-mut and ZE3 had lower inhibitory effects on viral infection compared to ZE2 (Fig. 6A). These results are consistent with their binding affinities with E2 glycoprotein. The intracellular HCV E2 protein and viral RNA levels were further determined by western blotting (Fig. 6B) and real-time RT-PCR analysis, respectively (Fig. 6C). Both the western blot and real-time RT-PCR showed that there was a dose-dependent inhibitory effect of the aptamer ZE2 on viral infection, and the inhibitory effect of 100 nM ZE2 aptamer was virtually equivalent to that of 500 IU IFN-α (Figs. 6B and 6C). These data illustrate that aptamer ZE2 may indeed block and inhibit viral infection of human hepatocytes.

## Discussion

This study shows that the ssDNA aptamers that bind specifically to HCV-E2 have been successfully generated by CS-SELEX for the first time. In contrast to most described SELEX methods, our approach relies on CS-SELEX to evolve aptamers for whole live cells that express a variety of surface markers representing molecular differences among different cells. The aptamers selected from CS-SELEX for whole live cells have much higher specificity than those aptamers from SELEX for a single target molecule. Most importantly, the aptamers we selected here bound to the natural form of the E2 envelope glycoprotein expressed on the mammalian CT26 cells, with glycosylation modifications that can affect protein folding, structure, and function. In addition, our approach involves DNA aptamers, which present several advantages over RNA aptamers, such as their increased stability, long shelf-life, and ease of chemical synthesis in large quantities at a relatively low cost.

Our finding firstly identified that the ZE2 ssDNA aptamer was able to identify the presence of HCV E2 surface antigen or free E2 antigen in HCV patient serum samples by a simple ELISA method. This method was highly consistent with the detectable results of HCV antibody and HCV RNA of HCV patient serum samples (Table 3, Fig. 4B). These data suggest that the ssDNA aptamer ZE2 could be used to diagnose early HCV infection by detecting the HCV surface antigen that is present in sera during the early stage of infection or before seroconversion. Furthermore, the ssDNA aptamer ZE2 could specifically target HCV envelope glycoprotein E2-expressing cells (Figs. 3B and 3C), and FITC-ZE2 seemed to be promising as a probe to detect HCV-infected cells. Such technology could be used both *in vitro* and *in vivo* for animal experiments in concert with fluorescence microscopy and optical *in vivo* imaging technology, respectively.

The limitations of the detection of anti-HCV antibodies or HCV RNA in serum currently used for the diagnosis of HCV infection have enhanced efforts to find a rapid, simple, sensitive, and specific alternative diagnostic approach to detect viral antigens. Similar to the detection of Hepatitis B Virus (HBV) surface antigen for the diagnosis of HBV infection, the ZE2 DNA aptamer may be helpful for the diagnosis of early HCV infection through a simple ELISA method to detect HCV E2 envelope surface antigen. Although HCV E2 contains 1∼2 amino acid sequence hypervariable regions (HVR) but most of the E2 residues, the chemicophysical properties and conformation of HVR are highly conserved [26]. CD81 is a conserved receptor for HCV E2 binding. The DNA aptamer ZE2 and CD81 share similar binding sites on HCV E2 (Fig. 5). Therefore, the DNA aptamer ZE2 holds great potential for the detection of HCV surface antigen in both clinical applications and basic research.

Based on genetic differences between HCV isolates, HCV has been classified into six genotypes (through 1–6) with several subtypes within each genotype (represented by letters) [27]. The preponderance and distribution of HCV genotypes varies globally. For example, in North America, genotype 1a predominates followed by 1b, 2a, 2b, and 3a. In Europe, genotype 1b is predominant followed by 2a, 2b, 2c, and 3a. Genotypes 4 and 5 are found almost exclusively in Africa [28]. The most common genotypes of HCV in China and Korea are subtypes 1b and 2a, followed by 1a or 6 [29], [30]. Genotype is clinically important in determining the potential patient response to interferon-based therapy and the required duration of such therapy. Our results showed that genotypes 1a, 1b, and 2a of E2 could be significantly captured by aptamer ZE2, and the binding affinities of aptamer ZE2 with genotypes 1a, 1b, and 2a of E2 were two-fold higher than those of genotypes 3, 4, 5, and 6 (Fig. 4D). Our experiments suggest that ZE2 can detect HCV particles in the sera of Chinese patients (Table 3). ZE2 may also hold great potential for use in the early diagnosis of HCV in Korea, the Americas, Europe, and Japan, and could also be helpful in determining the potential response to interferon-based therapeutic monitoring and prognostics.

Finally, we found that the DNA aptamer ZE2 could partially block HCV E2 binding to the CD81 receptor (Fig. 5F) and subsequent viral entry and infection of human hepatocytes (Fig. 6). These data suggest that DNA aptamer ZE2 may also hold potential for use as a drug against HCV infection, which is strengthened by its low immunogenicity and ease of chemical synthesis in large quantities at a relatively low cost. An anti-VEGF therapeutic aptamer was the first approved by the FDA for macular degeneration [31]. A second aptamer targeting the coagulation factor IXa is currently being tested as an anticoagulant in phase I/II clinical trials [32]. The newly selected aptamers, especially ZE2, are worthy of further clinical and basic research.

In summary, our data demonstrate that the selected aptamers, especially ZE2, hold great promise for developing new molecular probes as early diagnostic reagents or therapeutic drugs targeting HCV. Aptamers can also serve as tools for analyzing HCV-host cell interactions both *in vitro* and *in vivo*. The experimental strategy described in this study should be applicable for developing agonistic aptamers targeting a broad range of cell surface-expressed envelope glycoproteins of other viruses. The approach described here thus provides a new set of tools to screen early diagnostic reagents or therapeutic drugs against human viral diseases.

## Materials and Methods

### Cells and plasmids

Murine colon carcinoma cell line CT26 cells [33] were cultured in RPMI-1640 medium with 10% (v/v) fetal bovine serum (Hyclone). Rabbit anti-E2 polyclonal antibody was prepared as in our previous publication [33]. Human hepatocellular liver carcinoma cells Huh-7.5.1 [34] and HepG2 [35] were used in this study. HCV cell culture (HCVcc)-JFH-1 (2a) and Huh-7.5.1 cells were derived from Huh-7.5, which was kindly provided by Wen-Zhe Ho from the University of Pennsylvania School of Medicine [34]. Cloned E1E2 genes of HCV genotypes 1 through 6 in a pcDNA3 expression vector were kindly provided by Dr. Jonathan K. Ball from the University of Nottingham [36]. E1E2 gene stable-expressing HepG2 cells, E2 (genotype 1a) surface-expressing HepG2 (E2-HepG2), and CT26 (E2-CT26) stable cell lines were established by G418 selection [33]. *E. coli* BL21(DE3)/plysS and *E. coli* DH5α were used as described previously [37]. The bacteria were harvested by centrifugation at 13,000×g and suspended in sterile PBS at the appropriate concentration. Bacterial CFUs (colony forming units) were quantified from the absorbance at 600 nm. The CD81 recombinant protein was kindly provided by Dr. Zhongtian Qi of the Second Military Medical University in China [38].

### Serum samples

HCV serum samples or healthy serum samples were collected from 2006 to 2008 in Zhongnan Hospital of Wuhan University, Wuhan, China. The ethical approval for this study was obtained from the Zhongnan Hospital Research Ethics Committee. Written informed consents were obtained from all individuals.

### Getting the aptamers by Cell Surface- SELEX (CS-SELEX)

The CS - SELEX procedures were primarily based on our previous work [37], [39]. The library construction was designed, and random oligonucleotide templates were synthesized as a single-stranded 88-mer with the following sequence: 5′-GCGGAATTCTAATACGACTCACTATAGGGAACAGTCCGAGCC-N_30_-GGGTCAATGCGTCATA-3′, where the central N_30_ represents random oligonucleotides based on equal incorporation of A, G, C, and T at each position [37], [39]. The complementary strand was synthesized using the DNA polymerase I Klenow fragment with primer 1: 5′-GCGGGATCCTATGACGCATTGACCC-3′, where the underlined portion represents a *Bam*HI site. The initial dsDNA random library was generated by PCR amplification using primer 1 and primer 2: 5′-GCGGAATTCTAATACGACTCACTATAGGGAACAGT-3′, which contains an *Eco*RI site (underlined) [37], [39]. PCR reactions were performed as follows: a 100 µl of PCR mixture contained 10 µl of 10x PCR buffer, 0.2 mM dNTPs, 0.5 µM each primer, 10 nM template, and 2.5 U Taq DNA polymerase. The mixture was thermally cycled 30 times through 95°C for 1 min, 37°C for 30 s, and 58°C for 40 s, followed by a 5 min extension at 58°C. The ssDNA random library was then obtained by heating the dsDNA library at 85°C for 15 min and snap-cooling on ice for 3 min.

To initiate *in vitro* selection, random ssDNAs were incubated in binding buffer (25 mM Tris–HCl, 50 mM KCl, 200 mM NaCl, 0.2 mM EDTA, 5% (v/v) glycerol, and 0.5 mM DTT) for 30 min at 37°C, together with 10^6^ E2-CT26 cells. Following a wash with at least 40 column volumes of binding buffer, bound ssDNA was centrifuged at 12,000 rpm for 5 min, and the supernatant was discarded. The precipitant was diluted by adding 50 µL sterile ddH_2_O, boiled for 5 min, snap-cooled on ice for 3 min, extracted by phenol/chloroform (25∶24), and the supernatant was used as the template for PCR to obtain the ssDNA pool for the next round of selection. To remove nonspecifically bound DNA, we applied the counter-selection step using CT26 cells followed by the selection of E2-CT26 cells in each cycle. Each pool of aptamers was obtained by a different round of selection. The PCR products of each round were digested with *Eco*RI and *Bam*HI and then subcloned into pUC19. The bank was transformed into *E. coli* DH5α. Plasmid DNA was isolated from individual clones, purified, and analyzed by sequencing. Individual aptamers were obtained.

### Flow cytometry analysis

FITC-labeled aptamer pools or individual aptamers were obtained by PCR using FITC-labeled primers. Individual aptamers could also be synthesized according to their sequence. To demonstrate the binding specificity and characterize the binding parametersof the aptamers to E2 glycoprotein, FITC-labeled aptamers (250 nM) were incubated with 10^6^ E2-HepG2 (or E2-CT26), or HepG2 (or CT26) cells at 37°C for 15 min. They were then centrifuged at 2,000 rpm for 5 min, and the supernatant was discarded. The stained cells were then analyzed in a Beckman Coulter EPICS ALTRA II flow cytometer. The binding affinities of the individual aptamers to E2 glycoprotein-expressing cells were obtained by monitoring the mean fluorescence intensity of target E2-expressing cells bound to the FITC-labeled aptamers using a flow cytometer as described [40]. The concrete steps were the same as the flow cytometry analysis. The equation Y = BmaxX/(Kd+X) (Origin Pro 7.5) was used to calculate the target antigen-binding equilibrium dissociation constant Kd, where Bmax is the maximum percentage of fluorescence, Y is the mean percentage fluorescence, and X is the molecule mol concentration.

To detect whether the selected aptamer, ZE2, and CD81 share similar binding sites on the HCV E2 protein, 20 µg of recombinant CD81 proteins were incubated with 1×10^6^ E2-CT26 cells and then incubated with FITC-labeled aptamers (250 nM) at 37°C for 1 hr. The binding affinities between E2-CT26 cells and FITC-labeled aptamers were analyzed using flow cytometry. PE-, or FITC-conjugated rat IgG1 were used as an isotype-matched control.

### Capillary electrophoretic mobility shift assay (CEMSA)

GST*-*E2, or GST recombinant proteins were purified according to our previous publication [33]. In the analysis of the interaction between aptamers and E2 or GST proteins, CEMSA was performed on a Beckman P/ACE 5000 system (Fullerton, CA, USA). A series of mixtures with a fixed concentration of aptamers (250 nM) and increasing concentrations of E2, GST, or E2-GST recombinant proteins was prepared. The concentrations of the proteins in the mixtures were 0, 0.151, 0.302, 0.453, 0.604, and 0.755 µM, respectively. The mixtures were allowed to remain at equilibrium for 30 min at 4°C and were then injected using the pressure injection mode at 0.5 psi for 10 s.

### Fluorescence microscope imaging of E2-expressing cells bound with aptamers

The FITC-labeled aptamers (250 nM each) were incubated with 10^5^ E2-HepG2, or E2-CT26, or CT26 cells per well in 6-well plates with a cover slide in each well at 4°C overnight in the dark. The unbound aptamers in the supernatant were then discarded. The cell–ssDNA complex sediment was washed three times with PBS. Cells were then fixed with 4% paraformaldehyde and permeabilized with buffers containing 0.5% Triton X-100, 0.1% SDS, and 50 mmol/L Tris (pH 8.0). The fixed cells were stained with 100 µg/ml of the DNA-binding dye Propidium Iodide (PI) (Sigma) at room temperature for 5 min. The cells were washed three times with PBS. Imaging of the cells on the cover slide was performed with a confocal fluorescence microscope (Leica DM RXA) under 488 nm exciting light (green for FITC and red for PI) and visible light.

### Enzyme-Linked Immunosorbent Assay (ELISA)

A sandwich ELISA was performed for the virus capture assays using aptamers. The anti-E2 polyclonal antibody was coated into each well of a 96-well plate at 4°C overnight. After washing, the plates were blocked with 2% bovine serum albumin (BSA). After washing with phosphate-buffered saline (PBS)-0.1%(w/v) Tween-20 (PBST), 100 µL of the serum samples or HCVcc (4×10^5^ copies virus in 180 µl per well) was added to each well and incubated at 37°C for 30 min. HCV serum samples or healthy serum samples were collected from 2006 to 2008 (viral titer determined by real-time quantitative RT-PCR; provided by Zhongnan Hospital, Wuhan University, China). The plates were then washed, and the biotin-labeled aptamer (100 µL; 250 nM) was aliquoted into each well. After incubation at 37°C for 30 min, a 1∶1000 dilution of HRP-streptavidin was added and incubated at 37°C for 30 min. Finally, the samples were developed with a substrate solution containing o-phenylenediamine. The absorbance of each sample was measured at 450 nm.

To analyze the binding affinities between the aptamers and the E1E2 glycoproteins of different genotypes (genotypes 1 through 6), 96-well ELISA plates were pretreated by coating them with 100 µg/mL poly-L-lysine. After incubating at room temperature (RT) for 1 hr, the solution was removed, and the plates were rinsed with water and dried. Different genotypes of E1E2 gene stable-expressing HepG2 cells were established by G418 selection and coated in the 96-well plates. After washing, 100 µL of the biotin-labeled aptamers (250 nM) was added to each well. The same steps as described above were then followed.

### Measurement of anti-HCV antibody in the serum

Each fresh plasma sample was tested with an HCV antibody ELISA assay kit (KHB, Shanghai kehua Co. P. R. China), and HCV-positive samples (a sample-to-cut-off ratio (S/CO) higher than 1) were kept frozen until use.

### Measurement of inhibitory effects of aptamers on HCVcc infection by fluorescence microscopy analysis

JFH-1 HCVcc (JFH-1; 10 µL; 1×10^5^ copies of virus) was pre-incubated with the different doses of ssDNA aptamers at 37°C for 1 hr and then added to the 96-well plates with Huh-7.5.1 cells (4.5×10^5^/well) in DMEM with 10% FBS. After a 12 hr incubation, the virus-containing supernatant was removed and replaced by fresh media. Cells were incubated at 37°C for 72 hrs. Cells were washed with PBS and fixed for 20 min at room temperature in 4% paraformaldehyde. The cells were then washed with PBS containing 0.2% BSA. Fluorescence-labeled anti-E2 antibody was added to the Huh7.5.1 cells, incubated for 4 h, and washed with PBS three times. HCVcc-infected Huh7.5.1 cells were detected and measured by fluorescence microscopy.

### RNA quantification by real time fluorescence quantitative RT-PCR

Huh7.5.1 cells were cultured in a 6-well plate at a concentration of 5×10^5^ cells/well. Different concentrations of aptamers, aptamer mutants, or 1×10^4^ IU IFN-α were added to the cells along with 6×10^5^ viral copies of JFH-1 HCVcc. The mixtures were incubated at 37°C overnight. Cells were washed with DEPC-treated PBS to remove HCVcc in the supernatant, and total RNA was extracted from HCVcc-infected or non-infected Huh7.5.1 cells using Trizol reagent (Invitrogen Life Technologies); the RNA was then reverse transcribed using the First Strand cDNA synthesis kit (Fergment). The HCV primers were designed based on the HCV sequence (GenBank Accession # M67463) selected within the 5′-noncoding region (NCR) of the HCV genome according to our previous method [34]. RNA was quantitated by the real-time reverse transcription polymerase chain reaction (RT-PCR) using the QuantiTect^TM^ SYBR Green PCR Handbook Kit (QIAGEN) with the primers P1: 5′-CGGGAGAGCCATAGTGGTCTGCG-3′ (130∼152 nt) and P2: 5′-CTCGCAAGCACCCTATCAGGCAGTA-3′ (287∼311 nt) (i.e., specific for HCV), or the primers GAPDH-F: 5′–ACCACAGTCCATGCCATCAC–3′ and GAPDH-R: 5′–TCCACCACCCTGTTGCTGTA–3′ (i.e., specific for housekeeping gene GAPDH). The results were analyzed using Rotogene 6.0 software.

### Western blot analysis

Huh7.5.1 cells were cultured in a 6-well plate at a concentration of 5×10^5^ cells/well. Different concentrations of aptamers, aptamer mutants, or 500 IU IFN-α were added to the cells along with 6×10^5^ viral copies of JFH-1 HCVcc. The mixture was incubated at 37°C overnight, and cells were washed with PBS and lysed with 200 µl SDS-loading buffer. Samples were then boiled for 5 min and loaded onto a 12% SDS-polyacrylamide gel. After electrophoresis, proteins were transferred to a PVDF membrane, and E2 protein was detected with a specific anti-E2 polyclonal antibody; β-actin protein was detected with a specific anti-β-actin antibody.

### Statistical analysis

Experimental data were analyzed by ANOVA or an unpaired Student's *t* test. *P*-values<0.05 were considered statistically significant.
